# Association of Internet Addiction with Adolescents’ Lifestyle: A National School-Based Survey

**DOI:** 10.3390/ijerph18010168

**Published:** 2020-12-29

**Authors:** Chan Ying Ying, S Maria Awaluddin, Lim Kuang Kuay, Cheong Siew Man, Azli Baharudin, Ling Miaw Yn, Norhafizah Sahril, Mohd Azahadi Omar, Noor Ani Ahmad, Normala Ibrahim

**Affiliations:** 1 Centre for Family Health Research, Institute for Public Health, National Institutes of Health, Ministry of Health Malaysia, Setia Alam, Shah Alam 40170, Selangor, Malaysia; norhafizah_s@moh.gov.my; 2 Centre for Burden of Disease Research, Institute for Public Health, National Institutes of Health, Ministry of Health Malaysia, Setia Alam, Shah Alam 40170, Selangor, Malaysia; smaria@moh.gov.my; 3 Centre for Occupational Health Research, Institute for Public Health, National Institutes of Health, Ministry of Health Malaysia, Setia Alam, Shah Alam 40170, Selangor, Malaysia; limkk@moh.gov.my; 4 Centre for Nutrition Epidemiology Research, Institute for Public Health, National Institutes of Health, Ministry of Health Malaysia, Setia Alam, Shah Alam 40170, Selangor, Malaysia; smcheong@moh.gov.my (C.S.M.); ps_azlibaharudin@moh.gov.my (A.B.); 5 Centre for Non Communicable Diseases Research, Institute for Public Health, National Institutes of Health, Ministry of Health Malaysia, Setia Alam, Shah Alam 40170, Selangor, Malaysia; jane@moh.gov.my; 6 Sector for Biostatistics & Data Repository, Research Policy and Planning Division, National Institutes of Health, Ministry of Health Malaysia, Setia Alam, Shah Alam 40170, Selangor, Malaysia; drazahadi@moh.gov.my; 7 Director Office, Institute for Public Health, National Institutes of Health, Ministry of Health Malaysia, Setia Alam, Shah Alam 40170, Selangor, Malaysia; drnoorani@moh.gov.my; 8 Department of Psychiatry, Faculty of Medicine and Health Sciences, Universiti Putra Malaysia, Serdang 43400, Selangor, Malaysia; normala_ib@upm.edu.my

**Keywords:** internet addiction, adolescents, lifestyle, prevalence, Malaysia

## Abstract

Internet addiction (IA) among adolescents is an issue of growing concern with adverse effects on adolescents’ health and social functioning. This study aims to determine the prevalence of IA among school-going adolescents in Malaysia and its associated factors—specifically, lifestyle factors. A nationwide cross-sectional school-based health survey was conducted in 2017 among 27,497 students from 212 randomly selected secondary schools. Information regarding sociodemography, lifestyle, and internet use was obtained using a self-administered questionnaire. IA was measured using the Malay Version of Internet Addiction Test (MVIAT). The prevalence of internet addiction was 29.0%. A multivariable logistic analysis revealed that inadequate fruit and vegetable intakes, consumed carbonated soft drinks at least once a day, consumed fast food at least three days/week, sedentary behavior, current E-cigarette users, and ever/current alcohol drinkers were lifestyle factors significantly associated with IA. Adolescents from urban schools, of higher school grade, and those whose parents are married but living apart were also found to have a greater risk for internet addiction. A positive association was found between IA with unhealthy dietary and lifestyle behaviors among adolescents. The modification of lifestyle factors needs to be considered while developing strategies and interventions for awareness-raising and prevention of IA among adolescents.

## 1. Introduction

The internet has become one of the most important and useful technological tools available worldwide that has both positive and negative impacts in today’s modern society. It has become an essential part of our daily life, especially among youth and adolescents. The use of the internet is continually expanding. In 2020, there were almost 4.8 billion active internet users worldwide, equivalent to 62% of the global population. Asia was the region with the largest number of online users [1]. Excessive and uncontrollable use of the internet has led to the emergence of internet addiction (IA), which is defined as “excessive or poorly controlled preoccupations, urges or behaviors regarding computer use and internet access that lead to impairment or distress” [2]. Various types of online activities, such as online gaming, social networking, online gambling, online shopping, virtual sex, and information overload, are related to IA [3]. The eleventh revision of the International Classification of Diseases (ICD-11) released by the World Health Organization (WHO) in mid-2018 has included internet gaming disorder as a new disorder, which is considered an addictive behavior disorder that is often related to IA [4]. Therefore, research on IA has gained increasing attention from researchers, especially among adolescents.

Adolescents are considered more vulnerable to developing IA compared to other age groups [5]. Various studies on IA have predominantly focused on younger populations rather than the broader adult population. According to a recent review, the prevalence of severe problematic internet use/internet addiction ranged from 0% to 47.4%, while the prevalence of internet overuse/possible internet addiction ranged from 7.4% to 46.4% among students from Southeast Asia [6]. The wide range of prevalence rate could be attributed to different study designs, different assessment tools, or various diagnostic criteria, cultural backgrounds, and study samples [3,5]. Several tools for measuring IA have been developed. One of the most commonly used tools for assessment of IA is the Internet Addiction Test (IAT), which presented good internal consistency and concurrent validity [7]. 

According to the latest Internet Users Survey conducted by the Malaysian Communications and Multimedia Commission (MCMC) in 2018, the internet penetration rate has risen to 87.4% in 2018 compared to 76.9% in 2016. The age group of 20–24 years has been reported as the most frequent internet users [8]. As the internet grows in Malaysia, IA is emerging as a key problem, particularly among the younger generation there. In recent local studies, the prevalence of IA among adolescents and university students ranged from 23.0% to 43.0% using the validated Malay Version of the Internet Addiction Test (MVIAT) [9,10,11,12]. These statistics should make us aware that IA has become a significant health issue that requires attention among adolescents in Malaysia. Research indicated that adolescents with IA had a higher tendency for poor academic performance and high-risk behaviors, such as excessive alcohol use, illicit drug use, and tobacco use [13,14]. 

Recent research has revealed various factors of IA among adolescents, including sociodemographic [15], parental [16], psychosocial [17], and lifestyle factors [18]. Several studies revealed that IA has adverse effects on dietary behavior (i.e., frequent breakfast skipping, increased meal size, and snacking habit while using the internet) and lifestyle characteristics (i.e., less physically active, irregular sleep pattern, short sleep duration, and high use of alcohol and tobacco) among adolescents [18,19,20], which eventually influence the health of the young population. Poor dietary habits such as carbonated soft drinks and fast food consumptions have been reported to be related to IA among adolescents [21,22]. A recent study among young adults in Bangladesh also suggested that IA was significantly higher among respondents who had a smoking habit or did not involve in a considerable amount of physical activity [23]. However, studies focusing on IA and lifestyle among adolescents are still lacking in Malaysia. Thus far, the association between IA with lifestyle-related factors and high-risk behaviors has not been elucidated in Malaysia. Hence, given the recent rise of internet use among adolescents, this study aims to determine the prevalence of IA and its association with lifestyle factors, including dietary habits (fruit and vegetable intakes, carbonated soft drink intakes, and fast food intakes); physical activity; sedentary behavior; and the use of alcohol, tobacco, and drugs among school-going adolescents in Malaysia. 

## 2. Materials and Methods

### 2.1. Study Design and Participants

The current study used secondary data from a cross-sectional nationwide school-based survey, named the Adolescent Health Survey (AHS), conducted between March and May 2017 in Malaysia. The participants were school-going adolescents aged 13 to 17 years (Form 1 to Form 5; equivalent to 7th grade to 11th grade) studying in secondary schools. The national schools list obtained from the Ministry of Education Malaysia served as the school sampling frame. Both public and private schools were included in the sampling frame. A total of 212 secondary schools were chosen at random using a two-stage stratified cluster sampling design. The first stage of sampling was the selection of schools, while the second stage of sampling was the selection of classes in the selected schools. All students in the selected classes were eligible to participate in this survey. The detailed survey methodology was described in a published article by Awaluddin et al. [24]. 

Data were collected through a paper-based, structured, and self-administered questionnaire. The questionnaire was adapted from the Malaysian Global School-Based Student Health Survey (GSHS), which consists of ten modules on adolescent health, including lifestyle factors and risky behaviors [25]. Parental or guardian consent was obtained by distributing the consent form at one month prior to the actual survey. During the actual survey, informed consent was acquired, and proper instructions were given before the participants administered the questionnaires. This study was approved by the Medical Research and Ethics Committee (MREC), Ministry of Health Malaysia (NMRR-16-698-30042) and the Ministry of Education Malaysia. 

### 2.2. Measurements

#### 2.2.1. Malay Version Internet Addiction Test (MVIAT)

Internet addiction (IA) was measured using the validated Malay version of the Internet Addiction Test (MVIAT) [9]. The MVIAT consists of 20 items with a 5-point Likert scale of response (1 = rarely, 2 = occasionally, 3 = frequently, 4 = often, and 5 = always). The minimum score was 20, and the maximum score was 100. Respondents who scored 43 points and above were considered as having internet addiction. The MVIAT has been validated and has a good internal consistency (Cronbach’s alpha = 0.91, *p* < 0.001), parallel reliability (intraclass coefficient = 0.88, *p* < 0.001), and concurrent validity with the Compulsive Internet Use Scale (Pearson’s correlation = 0.84, *p* < 0.001) [9]. 

#### 2.2.2. Demographic Information

Demographic information such as age; sex; school locality (urban/rural); school grade (Form 1 to Form 5); ethnicity (Malays, Chinese, Indians, Bumiputera Sabah, Bumiputera Sarawak, and “Others”); and marital status of the parents (married and living together, married but living apart, or divorced/widower/separated/do not know) were obtained via a self-administered questionnaire. According to the schooling system in Malaysia, school grades for secondary schools refer to Form 1 to Form 5 (aged 13–17 years), which is equivalent to 7th grade to 11th grade in the American education system. “Others” included small minority groups that settled in Malaysia like Indonesians, Siamese, Portuguese, Burmese, and Bangladeshi. The demographics of the participants for the AHS were similar to those of general population of that age in Malaysia.

#### 2.2.3. BMI Measurements 

Height and weight of the students were measured by trained field workers using the calibrated SECA Portable Stadiometer 213 (SECA GmbH & Co. KG, Hamburg, Germany) and the TANITA Digital Weighing Scale HD 319 (TANITA Corp., Tokyo, Japan) to the nearest 0.1 cm and 0.1 kg, respectively. All measurements were taken twice, and the average value was used for data entry. The students’ body mass index (BMI)-for-age score was obtained by calculating the BMI-for-age z-scores using WHO AnthroPlus software. Students were classified into four categories: underweight (<−2SD z-scores), normal (≥−2SD to <+1SD z-scores), overweight (≥+1SD to <+2SD z-scores), and obese (≥+2SD z-scores) based on the WHO’s growth reference for children aged 5–19 years [26]. 

#### 2.2.4. Lifestyle Variables 

The following lifestyle variables (including dietary behavior and substance use) were used in the analysis: fruit and vegetable intakes; carbonated soft drink intakes; fast food intakes; physical activity status; sedentary behavior; and the use of alcohol, tobacco, and drugs. All these variables were based on the self-reported questionnaire from the Malaysian GSHS. 

#### 2.2.5. Dietary Behavior

Fruit intake was measured by the question “During the past 30 days, how many times per day did you usually eat fruit?” For measuring the vegetable intake, the question “During the past 30 days, how many times per day did you eat vegetables?” was asked. The Malaysian Dietary Guidelines (MDG) recommend that we should eat at least two servings of fruits and three servings of vegetables daily to make up the recommended five servings of fruits and vegetables daily [27]. The FAO/WHO expert consultation report recommends a minimum of 400 g of fruits and vegetables per day (excluding potatoes and other starchy tubers), an equivalent of ≥5 servings/day [28]. However, for the purpose of this study, and because the GSHS data provide only the frequency of the daily intake of fruits and vegetables with no reference to portion sizes, the consumption of fruits and vegetables ≥5 times/day was used as a general and proxy cut-off measure for the adequate intake of fruits and vegetables. Students who reported fruits intakes of less than two times per day and vegetable intakes of less than three times per day were considered as having an inadequate intake of fruits and vegetables.

Carbonated soft drink intake was measured by the question “During the past 30 days, how many times per day did you usually drink carbonated soft drinks such as Coca Cola, Sprite, and Pepsi? (Do not include diet soft drinks)”. Students who did not drink carbonated soft drinks during the past 30 days and consumed less than one time per day were categorized as “do not consume once a day”, while those who consumed one time or more per day were categorized as “consume at least once a day”. 

Fast food intake was measured by the question “During the past seven days, on how many days did you eat food from a fast food restaurant, such as McDonalds, KFC, and Pizza Hut?”, with response options set from 0 to 7 days. We categorized it into “do not consume fast food 3 days/week” and “consume fast food at least 3 days/week”.

#### 2.2.6. Physical Activity

Physical activity was measured by the question “During the past seven days, on how many days were you physically active for a total of at least 60 min per day?”. Responses were categorized into two groups: “active” and “inactive”. Students who reported physically active for at least 60 min per day on five to seven days in a week were considered physically active, while those being physically active on four or less days in a week were considered physically inactive. Sedentary behavior was measured by the question “How much time do you spend during a typical or usual day sitting and watching television, playing computer games, talking with friends, or doing other sitting activities (excluding the time spent sitting in school and for homework)?” Students that reported having at least three hours of sitting activities per day were classified as having sedentary behavior. 

#### 2.2.7. Substance Use

For tobacco use, a current cigarette smoker was identified based on the question “During the past 30 days, on how many days did you smoke cigarettes?” Current E-cigarette users were measured by the question “During the past 30 days, on how many days did you use e-cigarettes/vapes?” The response options for both questions ranged from 1 = 0 days to 7 = all 30 days. 

Alcohol drinking status was assessed based on two questions: (i) “How old were you when you had your first drink of alcohol?”, and (ii) “During the past 30 days, on how many days did you have at least one drink containing alcohol?” Alcohol drinking status was dichotomized as “ever/current” versus “never”. 

Drug use status was assessed based on two questions: (i) “During your life, how many times have you used drugs?”, and (ii) “During the past 30 days, how many times have you used drugs?” Drug use status was dichotomized as “ever/current” versus “never”.

### 2.3. Statistical Analysis

Statistical analysis was performed using the SPSS for Windows version 21.0 (IBM, New York, NY, USA). Apart from descriptive statistics of the demographic and lifestyle characteristics of participants, the prevalence of internet addiction was analyzed by demographic and lifestyle characteristics. Pearson’s chi-square test was performed to analyze the relationships between the study variables and IA. Univariable and multivariable logistic regression analyses were performed to look for the associated factors of IA. All variables with *p*-values < 0.25 in the univariable analysis were included in the final multivariable analysis model [29]. Odds ratios (OR) and 95% confidence intervals (CI) were presented as a measure of association. Statistical significance was set at *p* < 0.05. All data analyses took into account the complex sampling structure of survey data and sample weights.

## 3. Results

A total of 27,497 participants were involved in this survey, with an overall response rate of 89.2%. Of the 27,497 participants, 27,455 participants completed the questionnaire on the internet module, giving a response rate of 99.8% to the module.

Table 1 shows the demographic and lifestyle characteristics of the participants. There were 13,135 males (49.6%) and 14,362 females (50.4%) participating in this study. Slightly more than half (56.4%) of the participants were from urban schools, and 43.6% were from rural schools. The proportions of participants from Form 1 to Form 5 were almost equal, with about 20% in each Form. About two-thirds of the participants were Malays (63.1%). The majority of the participants’ parents were married and living together (82.2%). In terms of dietary behavior, most of the participants (76.5%) had inadequate intakes of fruits and vegetables (<five times per day), about one-third (36.9%) of the participants consumed soft drinks at least once a day, and about one in ten participants (11.1%) consumed fast food at least three days per week. The majority of the participants were physically inactive (80.2%), and half of the participants (50.1%) were sedentary (≥three hours of sitting activities per day). More than half of the participants (65.0%) had normal BMI, with small proportions who were underweight (6.5%), overweight (15.2%), and obese (13.3%). With regards to substance use, approximately one in ten of the participants were current cigarette smokers (13.8%) and current e-cigarette users (9.8%). About 20% of the participants were ever/current alcohol drinkers, and 4.3% of the participants were ever/current drug users. 

Table 2 shows the prevalence of internet addiction (IA) among Malaysian adolescents by demographic and lifestyle characteristics. The overall prevalence of IA was 29.0%. The bivariate analysis showed IA was significantly associated with demographic factors such as school locality, school grade, ethnicity, and parents’ marital status. Internet addiction was also significantly more prevalent among adolescents with unhealthy lifestyles, such as having inadequate intakes of fruits and vegetables (<five times/day), frequent fast food intakes (at least three days/week), being sedentary (≥three hours of sitting activities/day), smoking, alcohol drinking, and drug use. 

Table 3 shows the univariable and multivariable logistic regression analyses for IA among Malaysian adolescents. After adjusting for all other variables, the results from the multivariable logistic regression analysis revealed that urban adolescents (OR = 1.31; 95% CI: 1.16–1.49) were more likely to develop IA than rural adolescents. No significant association was observed between the students’ sex and IA. Students of higher school grades (Form 5) presented a two-fold greater risk for IA than those of lower school grades (Form 1). Adolescents whose parents were married but living apart were also more likely to be addicted to the internet (OR = 1.44; 95% CI: 1.16–1.78). Adolescents who were underweight were negatively associated with IA (OR = 0.83; 95% CI: 0.73–0.95), while adolescents who were overweight (OR = 1.03; 95% CI: 0.92-1.17) or obese (OR = 1.12; 95% CI: 0.99–1.26) were positively associated with IA; however, the positive association was not statistically significant. Several lifestyle factors such as inadequate fruit and vegetable intakes (OR = 1.21; 95% CI: 1.10–1.33), carbonated soft drink intakes of at least once a day (OR = 1.16; 95% CI: 1.07–1.26), fast food intakes of at least three days per week (OR = 1.40; 95% CI: 1.26–1.55), and sedentary behavior (OR = 2.44; 95% CI: 2.25–2.65) were significantly associated with IA among adolescents. Adolescents who were e-cigarette smokers (OR = 1.37; 95% CI: 1.20–1.57) and alcohol drinkers (OR = 1.20; 95% CI: 1.05–1.37) were also found to have a significantly higher risk for IA. 

## 4. Discussion 

In this study, we report the prevalence of internet addiction and its associated factors, including sociodemographic and lifestyle factors, in a large sample of school-going adolescents in Malaysia. 

The current survey demonstrated that about three in ten adolescents (29.0%) were addicted to the internet. In Malaysia, the prevalence of IA is expected to increase due to the rapid evolution of networking and the wide spread of internet coverage areas and the low cost of internet services, increasing usage of internet for educational and recreational activities, and widespread use of smartphones among adolescents [8]. Compared to the prevalence of problematic or addictive internet use reported from a study among adolescents aged 12–18 years in six Asian countries: China (19.3%), Hong Kong (34.6%), Japan (47.5%), South Korea (13.7%), Malaysia (37.5%), and the Philippines (50.9%) [30], the prevalence of IA among adolescents in our study (29.0%) was higher than that in China and South Korea but was lower than the others. The lower prevalence in our study compared to that reported for Malaysia in the study among six Asian countries was because of the use of different cut-off points for the Internet Addiction Test (IAT) in classifying IA. The study among six Asian countries also used another screening tool, the Revised Chen Internet Addiction Scale (CIAS-R), which reported a variation in prevalence of addictive internet use across the six Asian countries compared to those reported using the IAT [30]. The CIAS-R was found to contain more information than the IAT and might overestimate the prevalence of IA in adolescents [30]. Another recent study using the Malay version of the Internet Addiction Test (MVIAT) for IA among primary school children aged 11 years old in Malaysia reported a prevalence of 23.0% for IA [12]. In India, a recent study among 11th and 12th-grade students revealed that the prevalence of excessive internet use was 1.4%, while the prevalence of moderate and mild internet use was 30.3% and 23.9%, respectively [31]. Another recent study among adolescents aged 10-18 years in China reported a prevalence of 26.5% for IA [32]. It is worthwhile to note that the differences in the prevalences across different studies could be due to the usage of different tools, different definitions or criteria used for IA, different sample ages, and different cultural diversities and backgrounds of the populations studied. 

Several demographic factors were found to be associated with adolescents’ internet addiction. For instance, internet addiction was more common among urban school students. This is consistent with the findings from Sowndarya et al., where the problems of moderate and severe IA were found to be more common in urban areas than rural areas [33]. Moreover, this may be a direct reflection of the increased internet access for those from urban settings. Although many studies found that males were more likely than females to be internet addicts [32,33,34], our study showed no significant gender differences for IA among adolescents in Malaysia. There may be gender preferences with regards to the use of the internet. For instance, males were relatively more likely to engage in online gaming, entertainment, and leisure, while females were more likely to get involved in online shopping, blogging, Instagramming, and Facebooking [35]. However, both genders could be equally likely to use the internet and to be addicted to the internet. 

Consistent with findings from previous studies [32,36], our study showed that older adolescents tend to be addicted more than the younger students. Those of higher school grades demonstrated a higher risk of developing IA, possibly due to a higher level of independence at those ages, and children were given more privacy and less control from their parents [36]. It is also likely that older adolescents were the ones who used the internet for schoolwork, as well as for entertainment, more than other younger adolescents in this study. In addition, adolescents whose parents were married but living apart were more likely to develop internet addiction. A previous study found that children living in a single-parent household were significantly associated with internet addiction [37]. Children living with only their mother or their father may be more prone to internet addiction, possibly because of a lack of care from both parents. However, even within two-parent households, the quality of the parent-child relationship is an important factor in internet addiction [38].

A previous study reported a significant correlation between BMI and IA, whereby a higher BMI was found to be positively associated with IA [39]. Our study showed that adolescents who were overweight or obese were positively correlated with IA, but the results were not statistically significant. Our findings also revealed that adolescents who were underweight were significantly less likely to have IA. It was hypothesized that IA may indirectly result in a higher BMI, because adolescents who were internet addicts tended to be less physically active and more sedentary [39]. However, there are also other factors that could influence the BMI of adolescents, such as genetic predispositions and parental overweightness, which might affect the relationship between IA and BMI. This information was not investigated in this study. 

The current study clearly demonstrated that several dietary behaviors and lifestyle factors were significantly associated with internet addiction among adolescents. Unhealthy diets such as inadequate fruit and vegetable intakes, excessive carbonated soft drink intakes, and fast food intakes were positively associated with IA among adolescents. Previous studies pointed out that IA and time spent online were associated with disordered eating among adolescents [39,40]. For instance, those who became addicted to internet gaming frequently further restricted their eating habits to accommodate their playing. Food became secondary, meals were missed, and junk food and soft drinks consumed while playing online games. Our findings were supported by a study by Kamran et al., who indicated that fast food and fried items were the most consumed snacks, while carbonated beverages were the most consumed beverages among internet addicts [19]. 

In this study, it is not surprising that sedentary behavior (≥three hours of sitting activities a day) was strongly associated with IA among adolescents. The explanation is that adolescents who are addicted to the internet usually spend a long duration of time sitting at a device with the internet. Previous studies indicated that adolescents who were less physically active and obese were found to have higher IA rates [19,39,41]. It was hypothesized that IA may lead to less physical activity and indirectly result in obesity [39]. A multivariable logistic regression analysis showed that physical inactivity was associated with a greater risk of IA. However, the association was not statistically significant (*p* > 0.05). The physical activity data was based on self-reporting and may not accurately reflect the physical activity level of adolescents. Therefore, the association between physical activity and IA may not have been shown accurately. 

This study also reported that IA was associated with e-cigarette smoking and alcohol drinking. These findings were in accordance with Frangos et al., who reported that problematic internet use was associated with other potential addictive personal habits of smoking, drinking alcohol, or coffee and taking drugs among Greek university students [42]. Another study among Korean adolescents by Sung et al. also concluded that smoking, drinking, and drug abuse were associated with a greater risk of internet addiction [43]. However, in our study, the significant associations between the risk of IA with current cigarette smoking and drug use diminished in the final adjusted regression model. Current e-cigarette smokers were more likely to be internet addicts, probably because e-cigarettes are normally widely available through online platforms (e.g., social media and online stores), and people can easily access and purchase e-cigarettes via the internet [44]. 

Several limitations of the present study should be considered. First, the cross-sectional nature of the study prevented the determination of a causal relationship. Second, the utilization of self-reporting on internet addiction, as well as other lifestyle behaviors, may result in the possibility of a response bias. Third, not all possible factors (e.g., depression, anxiety, sleep disorders, aggressive behaviors, etc.) were included in the analysis. Moreover, data from long-term absentees were not reflected in the current analyses. Despite those limitations, the major strength of our study was its large nationwide representative sample of Malaysian school-going adolescents. Hence, the results of this study are generalizable to all school-going adolescents in Malaysia. The findings of this study provided important data on identifying the association between adolescents’ lifestyle and IA. Such information is important for public health decision-makers and healthcare practitioners to plan and develop appropriate intervention programs to control IA among adolescents. Future studies should attempt to determine the predictors of IA with more precise measurements on the various potential risk factors identified in this study. Longitudinal studies with a more accurate assessments of IA are also needed.

## 5. Conclusions

The study’s findings confirmed the positive associations between IA with unhealthy dietary and lifestyle behaviors among adolescents. The implications of our findings included the need for early screening, detection, and management of IA among adolescents, together with the cultivation of a healthy lifestyle (such as healthy eating, active living, and not smoking or drinking alcohol) to help in lowering the risk of IA. The modification of lifestyle factors needs to be considered while developing strategies and interventions for awareness-raising and the prevention of IA among adolescents. The relatively high prevalence of IA in this study should not only draw the attention of the parents but, also, warrant prompt action by the educationists and policymakers. Parents should be good role models and practice a healthy lifestyle themselves, because their children are much more likely to emulate these behaviors. Parents should also monitor their children’s online activities and limit internet use. As for educationists and policymakers, they play a key role in promoting and creating awareness on the importance of healthy lifestyles and healthy use of the internet among school children and the community, as well as developing guidelines regarding what should be the appropriate age for exposure to the internet, at what age a personal device for internet use should be provided to children, and permissible hours of internet use. 

## Figures and Tables

**Table 1:** Demographic and lifestyle characteristics of participants (*n* = 27,497).

	*n* (%)	95% CI
Sex		
Male	13,135 (49.6)	46.4–52.8
Female	14,362 (50.4)	47.2–53.6
Locality		
Urban	15,899 (56.4)	48.6–63.9
Rural	11,598 (43.6)	36.1–51.4
School grade		
Form 1	5704 (21.0)	19.7–22.4
Form 2	5501 (19.9)	18.3–21.5
Form 3	5837 (20.1)	18.6–21.6
Form 4	5532 (19.3)	17.7–21.0
Form 5	4923 (19.7)	17.5–22.2
Ethnicity		
Malays	18,713 (63.1)	58.7–67.3
Chinese	4100 (16.7)	13.3–20.7
Indians	1428 (7.0)	5.4–9.0
Bumiputera Sabah	1781 (7.0)	6.0–8.1
Bumiputera Sarawak	921 (4.5)	3.3–6.1
Others	554 (1.8)	1.3–2.4
Marital status of parents		
Married and living together	22,629 (82.2)	81.3–83.1
Married but living apart	915 (3.4)	3.1–3.8
Divorced/widower/separated/do not know	3900 (14.4)	13.6–15.2
Body mass index (BMI)		
Underweight (<−2SD)	1672 (6.5)	6.1–7.0
Normal (≥−2SD to < +1SD)	17,706 (65.0)	64.1–65.8
Overweight (≥+1SD to <+2SD)	4256 (15.2)	14.6–15.9
Obese (≥+2SD)	3677 (13.3)	12.6–14.0
Fruits and vegetables intake		
<5 times/day	21,206 (76.5)	75.0–77.8
≥5 times/day	6267 (23.5)	22.2–25.0
Carbonated soft drinks intake		
Do not consume once a day	17,635 (63.1)	61.2–65.0
Consume at least once a day	9827 (36.9)	35.0–38.8
Fast food intake		
Do not consume 3 days/week	24,407 (88.9)	88.0–89.8
Consume at least 3 days/week	3052 (11.1)	10.2–12.0
Physical activity status		
Active	5802 (19.8)	18.9–20.7
Inactive	21,609 (80.2)	79.3–81.1
Sedentary behavior (≥3 h of sitting activities)		
Yes	13,756 (50.1)	48.5–51.8
No	13,641 (49.9)	48.2–51.5
Current cigarette smoker		
Yes	3595 (13.8)	12.7–15.0
No	23,892 (96.2)	85.0–87.3
Current E-cigarette user		
Yes	2547 (9.8)	9.0–10.8
No	24,926 (90.2)	89.2–91.0
Alcohol drinking status		
Ever/current	4747 (19.3)	17.1–21.7
Never	22,736 (80.7)	78.3–82.9
Drug use status		
Ever/current	1020 (4.3)	3.6–5.1
Never	26,463 (95.7)	94.9–96.4

**Table 2:** Prevalence of internet addiction (% (95% CI)) among Malaysian adolescents (*n* = 27,455) by demographic and lifestyle characteristics.

Variables	Internet Addiction		*p*-Value (Chi-Square)
	Yes (*n* = 8049)	No (*n* = 19,406)	
Overall	29.0 (27.8–30.4)	71.0 (69.6–72.2)	
Sex			0.074
Male	29.9 (28.3–31.6)	70.1 (68.4–71.7)	
Female	28.2 (26.6–29.8)	71.8 (70.2–73.4)	
Locality			
Urban	32.2 (30.5–34.0)	67.8 (66.0–69.5)	<0.001
Rural	24.9 (23.1–26.7)	75.1 (73.3–76.9)	
School grade			<0.001
Form 1	18.4 (16.6–20.3)	81.6 (79.7–83.4)	
Form 2	23.5 (21.6–25.6)	76.5 (74.4–78.4)	
Form 3	31.4 (29.2–33.7)	68.6 (66.3–70.8)	
Form 4	34.8 (32.9–36.7)	65.2 (63.3–67.1)	
Form 5	37.9 (35.1–40.8)	62.1 (59.2–64.9)	
Ethnicity			0.008
Malays	28.2 (26.6–29.7)	71.8 (70.3–73.4)	
Chinese	34.3 (31.9–36.8)	65.7 (63.2–68.1)	
Indians	23.7 (20.3–27.5)	76.3 (72.5–79.7)	
Bumiputera Sabah	30.6 (24.6–37.4)	69.4 (62.6–75.4)	
Bumiputera Sarawak	29.0 (21.7–37.5)	71.0 (62.5–78.3)	
Others	25.6 (20.5–31.4)	74.4 (68.6–79.5)	
Marital status of parents			<0.001
Married and living together	28.5 (27.2–29.9)	71.5 (70.1–72.8)	
Married but living apart	37.0 (32.6–41.7)	63.0 (58.3–67.4)	
Divorced/widower/separated/do not know	30.3 (28.2–32.4)	69.7 (67.6–71.8)	
Body mass index (BMI)			0.063
Underweight (<−2SD)	25.2 (22.7–27.9)	74.8 (72.1–77.3)	
Normal (≥−2SD to < +1SD)	29.2 (27.8–30.6)	70.8 (69.4–72.2)	
Overweight (≥+1SD to <+2SD)	29.1 (27.0–31.3)	70.9 (68.7–73.0)	
Obese (≥+2SD)	30.0 (27.4–32.8)	70.0 (67.2–72.6)	
Fruits and vegetables intake			<0.001
<5 times/day	30.2 (28.9–31.6)	69.8 (68.4–71.1)	
≥5 times/day	25.1 (23.3–26.9)	74.9 (73.1–76.7)	
Carbonated soft drinks intake			0.107
Do not consume once a day	28.5 (26.9–30.1)	71.5 (69.9–73.1)	
Consume at least once a day	29.9 (28.4–31.5)	70.1 (68.5–71.6)	
Fast food intake			<0.001
Do not consume 3 days/week	28.1 (26.8–29.5)	71.9 (70.5–73.2)	
Consume at least 3 days/week	36.6 (34.3–39.0)	63.4 (61.0–65.7)	
Physical activity status			0.163
Active	30.2 (28.4–32.1)	69.8 (67.9–71.6)	
Inactive	28.8 (27.4–30.2)	71.2 (69.8–72.6)	
Sedentary behavior (≥3 h of sitting activities)			<0.001
Yes	39.2 (37.5–40.8)	60.8 (59.2–62.5)	
No	18.9 (17.8–20.1)	81.1 (79.9–82.2)	
Current cigarette smoker			<0.001
Yes	33.1 (30.6–35.8)	66.9 (64.2–69.4)	
No	28.4 (27.0–29.8)	71.6 (70.2–73.0)	
Current E-cigarette user			<0.001
Yes	37.7 (34.9–40.6)	62.3 (59.4–65.1)	
No	28.1 (26.8–29.5)	71.9 (70.5–73.2)	
Alcohol drinking status			<0.001
Ever/current	36.3 (33.8–38.9)	63.7 (61.1–66.2)	
Never	27.3 (26.0–28.7)	72.7 (71.3–74.0)	
Drug use status			<0.001
Ever/current	35.7 (31.8–39.8)	64.3 (60.2–68.2)	
Never	28.7 (27.4–30.1)	71.3 (69.9–72.6)	

**Table 3:** Univariable and multivariable logistic regression analyses for internet addiction among Malaysian adolescents by demographic and lifestyle characteristics.

Variables	Crude OR (95% CI)	*p*-Value	Adjusted OR ^a^ (95% CI)	*p*-Value
Sex				
Male	1.09 (0.99–1.20)	0.074	1.06 (0.96–1.18)	0.232
Female	1.00		1.00	
Locality				
Urban	1.44 (1.26–1.63)	<0.001	1.31 (1.16–1.49)	<0.001
Rural	1.00		1.00	
School grade				
Form 1	1.00		1.00	
Form 2	1.36 (1.16–1.60)	<0.001	1.29 (1.10–1.51)	0.002
Form 3	2.03 (1.74–2.37)	<0.001	1.84 (1.58–2.14)	<0.001
Form 4	2.36 (2.05–2.72)	<0.001	2.11 (1.83–2.43)	<0.001
Form 5	2.71 (2.29–3.21)	<0.001	2.33 (1.97–2.75)	<0.001
Ethnicity				
Malays	1.00		1.00	
Chinese	1.33 (1.17–1.52)	<0.001	1.03 (0.88–1.22)	0.698
Indians	0.79 (0.64–0.98)	0.029	0.71 (0.57–0.89)	0.004
Bumiputera Sabah	1.13 (0.82–1.54)	0.458	1.08 (0.84–1.40)	0.534
Bumiputera Sarawak	1.04 (0.70–1.54)	0.842	0.97 (0.68–1.38)	0.870
Others	0.88 (0.66–1.17)	0.369	0.84 (0.60–1.17)	0.306
Marital status of parents				
Married and living together	1.00		1.00	
Married but living apart	1.48 (1.22–1.79)	<0.001	1.44 (1.16–1.78)	0.001
Divorced/widower/separated/do not know	1.09 (0.99–1.20)	0.074	1.06 (0.96–1.17)	0.221
Body mass index (BMI)				
Underweight (<−2SD)	0.82 (0.72–0.93)	0.003	0.83 (0.73–0.95)	0.005
Normal (≥−2SD to < +1SD)	1.00		1.00	
Overweight (≥+1SD to <+2SD)	1.00 (0.89–1.12)	0.938	1.03 (0.92–1.17)	0.595
Obese (≥+2SD)	1.04 (0.93–1.17)	0.509	1.12 (0.99–1.26)	0.070
Fruits and vegetables intake				
<5 times/day	1.30 (1.18–1.42)	<0.001	1.21 (1.10–1.33)	<0.001
≥5 times/day	1.00		1.00	
Carbonated soft drinks intake				
Do not consume once a day	1.00		1.00	
Consume at least once a day	1.07 (0.99–1.17)	0.107	1.16 (1.07–1.26)	0.001
Fast food intake				
Do not consume 3 days/week	1.00		1.00	
Consume at least 3 days/week	1.48 (1.34–1.63)	<0.001	1.40 (1.26–1.55)	<0.001
Physical activity status				
Active	1.00		1.00	
Inactive	0.94 (0.85–1.03)	0.163	1.02 (0.91–1.13)	0.771
Sedentary behavior (≥3 h of sitting activities)				
Yes	2.76 (2.53–3.01)	<0.001	2.44 (2.25–2.65)	<0.001
No	1.00		1.00	
Current cigarette smoker				
Yes	1.25 (1.11–1.41)	<0.001	1.01 (0.88–1.15)	0.944
No	1.00		1.00	
Current E-cigarette user				
Yes	1.55 (1.37–1.75)	<0.001	1.37 (1.20–1.57)	<0.001
No	1.00		1.00	
Alcohol drinking status				
Ever/current	1.52 (1.35–1.71)	<0.001	1.20 (1.05–1.37)	0.009
Never	1.00		1.00	
Drug use status				
Ever/current	1.38 (1.16–1.64)	<0.001	1.13 (0.88–1.46)	0.345
Never	1.00		1.00	

OR, odds ratio and CI, confidence interval. ^a^ Odds ratios adjusted for all other variables with a crude analysis *p*-value of less than 0.25. Note: Multicollinearity and interactions were checked and not found. Classification table = 71.0%. Nagelkerke Pseudo-R-squared = 0.111.
